# Anti-Seizure Medication Use Before Electroencephalography in Infants

**DOI:** 10.1001/jamanetworkopen.2025.51124

**Published:** 2025-12-23

**Authors:** Nathalia Beller, Madeline Fields, Chad H. Hogan, Sushma Krishna, Benjamin S. Glicksberg, Courtney E. Juliano, Felix Richter

**Affiliations:** 1 Department of Genetics, Icahn School of Medicine at Mount Sinai, New York, New York; 2 Department of Neurology, Icahn School of Medicine at Mount Sinai, New York, New York; 3 Division of Neonatology, Department of Pediatrics, Icahn School of Medicine at Mount Sinai, New York, New York; 4 Center for AI in Children’s Health, Icahn School of Medicine at Mount Sinai, New York, New York; 5 Children’s Hospital of Philadelphia, Philadelphia, Pennsylvania

## Abstract

This cross-sectional study examines antiseizure medication use before electroencephalography in infants in the neonatal intensive care unit population.

## Introduction

Seizures are among the most common neurologic emergencies in infants. These events are often subtle and may be mistaken for normal movements.^1^ Accurate seizure identification is crucial, as untreated seizures may cause brain injury, while unnecessary treatment exposes infants to the risks of antiseizure medications (ASMs), including drug toxicity and adverse development.^2^

Video electroencephalography (EEG) is the reference standard for diagnosing infant seizures, yet its cost and need for specialized expertise limit widespread use.^3^ Understanding care gaps from restricted video EEG availability is critical for improving seizure management, where delays impact neurodevelopment. Although prior studies assessed treatment delays after EEG initiation,^4^ none have examined ASM use before confirmation with reference standard video EEG. This study provides data on ASM administration based on clinical suspicion alone, associated neuropathology, the care setting where seizures were first suspected, and outcomes. We included infants outside the neonatal period to characterize this growing neonatal intensive care unit (NICU) population.

## Methods

Clinical data were retrospectively collected between February 2021 and December 2022 for 115 infants who met inclusion criteria of being younger than 1 year and undergoing video EEG monitoring. This cross-sectional study followed the STROBE reporting guideline. The Mount Sinai institutional review board approved a waiver of consent to conduct this study. Statistical analyses were conducted using R version 4.3.1 (R Project for Statistical Computing). A Fisher exact test was used for all categorical comparisons. Odds ratios (ORs) with 95% CIs were calculated for 2 × 2 contingency tables. Statistical significance was set at *P* < .05; all tests were 2-sided. Additional information can be found in the eMethods in Supplement 1.

## Results

We included 115 infants who underwent video EEG (median [IQR] age, 6 [1-49] days; 62 female [53.9%]), of whom 46 (40%) received loading doses of at least 1 ASM (Table). Of these 46 infants, 27 (59%) received ASMs before video EEG, while 19 (41%) received ASMs only after video EEG initiation. Among the 27 infants treated before EEG, 24 (89%) had epileptiform activity, including 14 (52%) with seizures subsequently observed on EEG (Figure). The remaining 13 infants (48%) without electrographic seizures likely mark the upper bound on overtreatment from clinical signs alone.

**Table.** Demographic and Clinical Characteristics of All Study Infants

Descriptive factor	Patient, No. (%)				
	Before vEEG (n = 27)	After vEEG (n = 19)	Infants with ASM load (n = 46)	Infants without ASM load (n = 69)	All infants (n = 115)
Gestational age at birth, median (range), wk	36.9 (24.6-41.1)	37.8 (30.4-40.1)	37.3 (24.1-41.1)	36.3 (23.0-41.0)	36.7 (23.0-41.1)
Age in months of first ASMs administration					
<1	20 (74.1)	14 (73.7)	34 (73.4)	43 (62.3)	77 (67.0)
1-3	5 (18.5)	4 (21.0)	9 (19.6)	13 (18.8)	22 (19.1)
3-6	1 (3.7)	0	1 (2.2)	8 (11.6)	9 (7.8)
≥6	1 (3.7)	1 (5.2)	2 (4.3)	5 (7.2)	7 (6.1)
Sex					
Female	12 (44.4)	7 (36.8)	19 (41.3)	34 (49.3)	53 (46.1)
Male	15 (55.6)	12 (63.2)	27 (58.7)	35 (50.7)	62 (53.9)
Race					
American Indian or Alaskan	2 (7.4)	2 (10.5)	4 (8.7)	1 (1.4)	5 (4.3)
Asian	2 (7.4)	1 (5.2)	3 (6.52)	7 (10.1)	10 (8.7)
Black or African American	5 (18.5)	4 (21.5)	9 (19.6)	18 (26.1)	27 (23.5)
Native Hawaiian or Pacific Islander	0	0	0	1 (1.4)	1 (0.9)
White	6 (22.2)	8 (42.1)	14 (30.4)	17 (24.6)	31 (27.0)
Other^a^	8 (29.6)	3 (15.8)	11 (23.9)	22 (31.9)	33 (29.0)
Unknown	4 (14.8)	1 (5.2)	5 (10.9)	3 (4.3)	8 (7.0)
Ethnicity					
Hispanic or Latino	9 (33.3)	4 (21.5)	13 (28.3)	21 (30.4)	34 (29.6)
Non-Hispanic	11 (40.7)	12 (63.2)	23 (50.0)	35 (50.7)	58 (50.4)
Unknown	7 (25.9)	3 (15.8)	10 (21.7)	13 (18.8)	23 (20.0)
Neurologic pathology					
Hypoxic ischemic encephalopathy	4 (14.8)	7 (36.8)	11 (23.9)	14 (20.3)	25 (21.7)
Genetic or idiopathic epilepsy	5 (18.5)	5 (26.3)	10 (21.7)	4 (5.8)	14 (12.2)
Intraventricular hemorrhage	5 (18.5)	0	5 (10.9)	2 (2.9)	7 (6.1)
Stroke	3 (11.1)	2 (10.5)	5 (10.9)	0	5 (4.3)
Structural brain malformation	2 (7.4)	2 (10.5)	4 (8.7)	1 (1.4)	5 (4.3)
Other	5 (18.5)	3 (15.8)	8 (17.4)	7 (10.1)	15 (13.0)
None	3 (11.1)	0	3 (6.5)	41 (59.4)	44 (38.3)

Abbreviations: ASM, antiseizure medication; vEEG, video electroencephalography.

^a^ Other was the option caregivers could select if they did not self-identify with a prespecified racial category.

**Figure.**
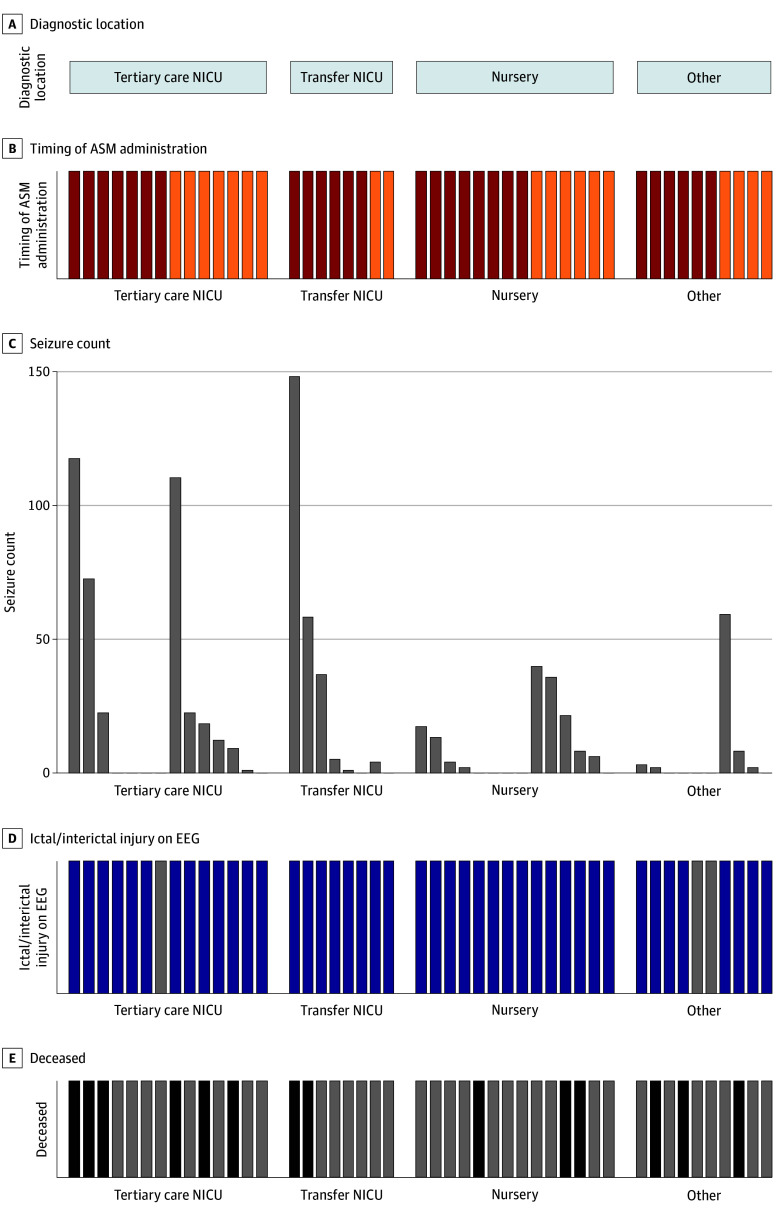
Seizure Detection Among the 46 Infants Who Received ASM Loading Doses and Video Electroencephalography (EEG) (A) Initial clinical context in which a concerning neurological finding or seizure risk factor was first observed. Notably, 17.8% occurred at a neonatal intensive care unit (NICU) without video EEG (transfer NICU), 31.1% occurred in the well-baby nursery, and 20.0% were observed in other contexts (either at home or in the emergency department). (B) Timing of antiseizure medication (ASM) administration relative to when video EEG was initiated. Red represents infants who received ASM prior to video EEG hookup. (C) Number of seizures detected on video EEG per infant. (D) Presence of epileptiform activity on video EEG (dark blue) indicating ictal or interictal injury, which could reflect prior seizure activity or proclivity to future seizures. (E) Mortality, with black depicting infants who died.

The distribution of neuropathology was diverse (Table). All infants with intraventricular hemorrhage (IVH) received ASMs before EEG (OR, 0; 95% CI, 0-1.5; *P* = .07), while infants with hypoxic ischemic encephalopathy (HIE) more often received ASMs on EEG (OR, 3.3; 95% CI, 0.7-18.4; *P* = .15). Video EEG was protocolized for HIE, but 36% (4 of 11) still received ASMs before EEG. Mortality was high among all infants who received ASMs (14 of 46 [30%]) and similar if ASMs were administered before vs after starting video EEG (OR, 0.9; 95% CI, 0.2-4.0; *P* > .99), highlighting their acuity.

## Discussion

This study quantified the high rate of ASM decisions without EEG. ASMs were often initiated on clinical suspicion alone, and most EEGs confirmed epileptiform activity, suggesting low false positive rates by examination and that treatment benefits often outweigh risks. NICU staff could be less conservative with ASMs before EEG; treating before EEG occurred frequently with IVH. Our data supported clinical judgement in other contexts, including HIE before protocolized video EEG. Results are limited to a single network, and regional practice, transfer timing, and video EEG availability could influence treatment thresholds. Generalizability should be evaluated in other networks. The frequency of pre-EEG ASM administration emphasizes the need for scalable diagnostic tools. Algorithmic decision support is already used with video EEG^5^; portable EEG could provide similar support in NICUs without video EEG. Computer vision with video could further augment EEG and remove artifacts, recognize paroxysmal events, or classify semiologies,^6^ especially with rare genetic epilepsies. Further research is needed to refine seizure identification for timely interventions, reduce strain on neurologic emergency transport systems, and ultimately improve infant outcomes.

## Notes

Supplement 1.
eMethods.

Supplement 2.
Data Sharing Statement
