# Supplementary material for: Anti-Seizure Medication Use Before Electroencephalography in Infants
Source: JAMA Netw Open. 2025 Dec 23;8(12):e2551124. doi: 10.1001/jamanetworkopen.2025.51124 (PMC12728646; doi:10.1001/jamanetworkopen.2025.51124)
Supplement: Supplement 1. — eMethods. [file jamanetwopen-e2551124-s001.pdf]

Supplemental Online Content

Beller N, Fields M, Hogan CH, et al. Anti-seizure medication use before electroencephalography in infants. *JAMA Netw Open*. 2025;8(12):e2551124. doi:10.1001/jamanetworkopen.2025.51124

eMethods.

This supplemental material has been provided by the authors to give readers additional information about their work.

© 2025 Beller N et al. *JAMA Network Open*.

eMethods.

Our study was conducted and reported in accordance with the Strengthening the Reporting of Observational Studies in Epidemiology (STROBE) guidelines for cross-sectional studies to provide a standardized assessment of ASM use without EEG support in infants. Our findings were ascertained at a tertiary pediatric hospital with both in-born and out-born infants. Although the cross-sectional nature of our study with broad inclusion criteria provides a generalizable snapshot of pre-EEG ASM administration, selection bias with regards to the proportion of in-born vs out-born infants may affect the applicability of these results to other centers with different proportions of in-born infants, referral patterns, or neurologic resources.

Statistical methods

Statistical analyses were conducted using R, version 4.3.1 (R Foundation for Statistical Computing, Vienna, Austria). Descriptive statistics included medians and ranges for continuous variables and counts and percentages for categorical variables. Fisher's exact test was used for all categorical comparisons. Odds ratios (ORs) with 95% CIs were calculated for 2 × 2 contingency tables. All *P* values were 2-sided, with significance set at $P < .05$.

© 2025 Beller N et al. *JAMA Network Open*.
