# Supplementary material for: Anti-Seizure Medication Use Before Electroencephalography in Infants
Source: JAMA Netw Open. 2025 Dec 23;8(12):e2551124. doi: 10.1001/jamanetworkopen.2025.51124 (PMC12728646; doi:10.1001/jamanetworkopen.2025.51124)
Supplement: Supplement 2. — Data Sharing Statement [file jamanetwopen-e2551124-s002.pdf]

Data Sharing Statement

Beller. Anti-Seizure Medication Use Before Electroencephalography in Infants. *JAMA Netw Open*. Published December 23, 2025. doi:10.1001/jamanetworkopen.2025.51124

Data
Data available: Yes
Data types: Deidentified participant data
How to access data: The datasets used and/or analyzed during the current study are available from the corresponding author on reasonable request.
When available: With publication

Supporting Documents
Document types: None

Additional Information
Who can access the data: The datasets used and/or analyzed during the current study are available from the corresponding author on reasonable request.
Types of analyses: For any purpose.
Mechanisms of data availability: The datasets used and/or analyzed during the current study are available from the corresponding author on reasonable request.
Any additional restrictions: None.
